# Staphylococcus aureus Panton-Valentine Leukocidin worsens acute implant-associated osteomyelitis in humanized BRGSF mice

**DOI:** 10.1093/jbmrpl/ziad005

**Published:** 2024-01-04

**Authors:** Marloes I Hofstee, Claudia Siverino, Motoo Saito, Himanshu Meghwani, James Tapia-Dean, Samson Arveladze, Maria Hildebrand, Javier Rangel-Moreno, Martijn Riool, Stephan Zeiter, Sebastian A J Zaat, T Fintan Moriarty, Gowrishankar Muthukrishnan

**Affiliations:** AO Research Institute Davos, 7270 Davos, Switzerland; Department of Medical Microbiology and Infection Prevention, Amsterdam UMC, Amsterdam institute for Infection and Immunity, University of Amsterdam, 1105 AZ Amsterdam, The Netherlands; AO Research Institute Davos, 7270 Davos, Switzerland; Center for Musculoskeletal Research, University of Rochester Medical Center, Rochester, NY 14642, United States; Department of Orthopaedics and Rehabilitation, University of Rochester Medical Center, Rochester, NY 14618, United States; Center for Musculoskeletal Research, University of Rochester Medical Center, Rochester, NY 14642, United States; Department of Orthopaedics and Rehabilitation, University of Rochester Medical Center, Rochester, NY 14618, United States; AO Research Institute Davos, 7270 Davos, Switzerland; AO Research Institute Davos, 7270 Davos, Switzerland; AO Research Institute Davos, 7270 Davos, Switzerland; Division of Allergy, Immunology and Rheumatology, Department of Medicine, University of Rochester Medical Center, Rochester, NY 14620, United States; Department of Medical Microbiology and Infection Prevention, Amsterdam UMC, Amsterdam institute for Infection and Immunity, University of Amsterdam, 1105 AZ Amsterdam, The Netherlands; Department of Trauma Surgery, University Hospital Regensburg, 93053 Regensburg, Germany; AO Research Institute Davos, 7270 Davos, Switzerland; Department of Medical Microbiology and Infection Prevention, Amsterdam UMC, Amsterdam institute for Infection and Immunity, University of Amsterdam, 1105 AZ Amsterdam, The Netherlands; AO Research Institute Davos, 7270 Davos, Switzerland; Center for Musculoskeletal Research, University of Rochester Medical Center, Rochester, NY 14642, United States; Department of Orthopaedics and Rehabilitation, University of Rochester Medical Center, Rochester, NY 14618, United States; Department of Microbiology and Immunology, University of Rochester Medical Center, Rochester, NY 14642, United States

**Keywords:** Humanized mice, neutrophils, osteomyelitis, staphylococcus aureus, PVL

## Abstract

*Staphylococcus aureus* is the most common pathogen that causes implant-associated osteomyelitis, a clinically incurable disease. Immune evasion of *S. aureus* relies on various mechanisms to survive within the bone niche, including the secretion of leukotoxins such as Panton-Valentine leukocidin (PVL). PVL is a pore-forming toxin exhibiting selective human tropism for C5a receptors (C5aR1 and C5aR2) and CD45 on neutrophils, monocytes, and macrophages. PVL is an important virulence determinant in lung, skin and soft tissue infections. The involvement of PVL in *S. aureus* pathogenesis during bone infections has not been studied extensively yet. To investigate this, humanized BALB/c Rag2^−/−^Il2rg^−/−^Sirpa^NOD^Flk2^−/−^ (huBRGSF) mice were subjected to transtibial implant-associated osteomyelitis with community-acquired methicillin-resistant *S. aureus* (CA-MRSA) USA300 wild type strain (WT), an isogenic mutant lacking *lukF/S-PV* (Δpvl), or complemented mutant (Δpvl+pvl). Three days post-surgery, Δpvl-infected huBRGSF mice had a less severe infection compared to WT-infected animals as characterized by 1) improved clinical outcomes, 2) lower ex vivo bacterial bone burden, 3) absence of staphylococcal abscess communities (SACs) in their bone marrow, and 4) compromised MRSA dissemination to internal organs (liver, kidney, spleen, heart). Interestingly, Δpvl-infected huBRGSF mice had fewer human myeloid cells, neutrophils, and HLA-DR^+^ monocytes in the bone niche compared to WT-infected animals. Expectedly, a smaller fraction of human myeloid cells were apoptotic in the Δpvl-infected huBRGSF animals. Taken together, our study highlights the pivotal role of PVL during acute implant-associated osteomyelitis in humanized mice.

## Introduction

*S. aureus* is an opportunistic human-adapted pathogen using various mechanisms to survive within the host and cause persistent infections. During implant-associated osteomyelitis, *S. aureus* employs several strategies to persist within the host. *S. aureus* could grow as biofilm on implants or on bone sequestra.^(^^1^^,^^2^^)^ It initially forms a staphylococcal abscess community (SAC) that subsequently triggers purulent abscess formation.^(^^3–7^^)^ Additionally, the bacterium hides in osteocyte-lacuno canalicular networks (OLCNs)^(^^6–9^^)^ or intracellularly in cells like osteoblasts,^(^^10^^)^ osteoclasts,^(^^11^^)^ osteocytes,^(^^12^^)^ and phagocytes.^(^^13^^)^ Another survival strategy of *S. aureus* is the secretion of pore-forming toxins such as leukotoxins, which cause cell lysis by affecting the membranal integrity of leukocytes.^(^^14^^)^

Panton-Valentine leukocidin (PVL) is a bi-component staphylococcal leukotoxin, which mainly targets and lyses neutrophils, monocytes, and macrophages by binding the complement receptors C5aR1 and C5aR2 with its S-component^(^^15^^)^ and CD45 with its F-component.^(^^16^^)^ PVL-producing *S. aureus* strains are commonly present in skin and soft tissue infections (SSTIs) and more severe diseases, including necrotizing pneumonia, necrotizing fasciitis, muscular abscesses, and sepsis.^(^^17–19^^)^ Moreover, PVL-secreting *S. aureus* strains cause severe and difficult-to-treat osteomyelitis in humans.^(^^20^^,^^21^^)^ In contrast, clinical and animal studies (mice or rabbits) propose PVL-positive *S. aureus* strains to have a similar or a less virulent phenotype compared to PVL-lacking *S. aureus* strains in lung, skin and soft tissue infections.^(^^22–25^^)^

PVL has host-specific activities, which may partially explain these controversial results. Specifically, PVL can lyse human and rabbit innate immune cells but has no activity towards murine innate immune cells.^(^^15^^)^ Interestingly, PVL activity is dose-dependent: high PVL doses promote pore formation, and lower doses activate innate immune cells, which may benefit the host by shaping antibacterial responses.^(^^26^^,^^27^^)^

Recent studies showed that humanized mice infected with an *S. aureus* strain lacking PVL developed less severe pneumonia 1 day post-inoculation or SSTIs symptoms 3 days post-infection. Thus, it was concluded that *S. aureus* pathogenesis depends on PVL.^(^^28^^,^^29^^)^ However, whether PVL is critical to *S. aureus* pathogenesis during osteomyelitis in humanized mice remains to be investigated. We recently developed a humanized mouse model of implant-associated osteomyelitis to account for human-specific *S. aureus* pathogenesis during bone infections.^(^^30^^)^ We demonstrated that humanized mice have: 1) increased weight loss, SACs, and extensive osteolysis during MRSA infection, and 2) increased susceptibility to osteomyelitis-induced sepsis.^(^^30^^)^

In this study, we assessed if PVL is required for the virulence of CA-MRSA USA300 strain AH-LAC during acute osteomyelitis in humanized BALB/c Rag2^−/−^Il2rg^−/−^Sirpa^NOD^Flk2^−/−^ mice (huBRGSF). HuBRGSF with functional human neutrophils and monocytes/macrophages^(^^31^^,^^32^^)^ were generated by engrafting BRGSF animals with human CD34^+^ hematopoietic stem cells (HSC). Here, we utilized WT, an isogenic USA300 mutant lacking *lukF/S-PV* (Δpvl), and a complemented mutant strain (Δpvl+pvl)^(^^25^^,^^33^^)^ to perform the bone infection studies. These in vivo studies had a duration of 3 days as previous studies with humanized mice showed that PVL contributes to *S. aureus* virulence in the early stages of infection^(^^28^^,^^29^^)^ and given that the innate immune cells targeted by PVL are known to infiltrate into the site of infection during the acute phase of an osteomyelitis.^(^^34–36^^)^ Bacterial load (colony forming units; CFUs) within the operated limb and organs, human immune cell abundance, and bacterial structures in bone marrow showed that Δpvl-infected huBRGSF had a less severe bone infection than WT-infected huBRGSF. These results indicate that the leukotoxin PVL does facilitate *S. aureus* pathogenicity during acute implant-associated osteomyelitis in humanized mice.

## Materials and methods

### Bacterial strains and culture

The following bacterial strains were used in this study: the community-acquired methicillin-resistant *S. aureus* (CA-MRSA) isotype USA300 AH-LAC as wild type strain (WT), the *lukF/S-PV* isogenic mutant strain USA300 AH-LAC *lukS/F-PV* (Δpvl),^(^^25^^)^ and the USA300 AH-LAC Δpvl trans-complemented mutant for PVL referred to as LUG1515 (Δpvl+pvl),^(^^33^^)^ all a kind gift from prof. Gerard Lina (CIRI, Lyon). Overnight cultures were grown in tryptic soy broth (TSB; Oxoid, Basel, Switzerland) at 37°C with shaking. The USA300 Δpvl+pvl strain cultures were supplemented with 10 μg/ml chloramphenicol to ensure plasmid maintenance.

Planktonic growth of the three above mentioned strains was assessed by diluting overnight cultures from the strains 1:1000 in fresh TSB, pipetting 200 μl of these diluted cultures in triplicate in a 96-wells plate, and measuring the turbidity of these culture at 600 nm using a microtiter-plate reader (MULTISKAN GO; Thermo Fisher Scientific, Basel, Switzerland) for 12 h at 37°C.

Biofilm from the USA300 WT, Δpvl, or Δpvl+pvl strains were generated by resuspending the strains to a 10^6^ CFU/ml concentration in TSB with 1% glucose (Sigma-Aldrich, Buchs, Switzerland) and pipetting 200 μl of this inoculum in a 96-flat bottom well plate followed by a 24 h incubation at 37°C. TSB with 1% glucose was used as a blank control. Thereafter, wells were washed with PBS, fixed with 70% methanol for 20 min, air-dried for 5 h and stained with crystal violet (Sigma-Aldrich) for 15 min.^(^^37^^)^ The staining solution was removed, wells were filled with 95% ethanol, and the optical density of the crystal violet-stained biofilm was measured at 595 nm using a microtiterplate reader (MULTISKAN GO; Thermo Fisher Scientific). OD measurements of biofilm were adjusted based on the average OD of the blank + (3 × SD of the blank), being the OD cut-off value, to obtain corrected OD values.^(^^37^^)^

In vitro SACs of the USA300 WT, Δpvl, or Δpvl+pvl strains were generated as described earlier.^(^^38^^)^ In short, 40 μl collagen gel (Gibco, Basel, Switzerland) was used as base, then 25 μl bacterial inoculum (approximately 14 CFUs) was pipetted on top and covered with 100 μl collagen gel and, lastly, this was supplemented with 600 μl human plasma (Regional Blood Donation Service SRK Graubünden, Chur, Switzerland). Phase contrast images were taken with the Zeiss Axio Vert.A1 microscope (Zeiss, Oberkochen, Germany). For CFU quantification, samples, together with 1 mm zirconium oxide beads (Next Advance, New York, United States) and 250 μl phosphate buffered saline (PBS; Gibco), were homogenized with the Bullet Blender (Next Advance) for 3 min (speed 10) and sonicated (Bandelin electronic, Berlin, Germany) for 3 min at 35 kHz. Serial dilutions of the homogenized samples were prepared, and 10 μl smears of these dilutions were plated in triplicate on tryptic soy agar (TSA; Oxoid) plates, which were incubated for 24 h at 37°C.

To inoculate stainless-steel pins (4 mm long with a cross-section of 0.2 mm × 0.5 mm and bend at 1 mm to form an L-shape) with approximately 5 × 10^5^ CFUs or 5 × 10^4^ CFUs of either the USA300 WT, Δpvl, or Δpvl+pvl strains, pins were submerged in 1 ml undiluted or 1:10 diluted (in sterile PBS) overnight culture for 20 min, and subsequently removed from the liquid, placed into a sterile petri-dish, and air-dried for 5 min. Some pins were used as implants for the in vivo studies and others were directly processed for CFU quantification. This was done by sonicating the pins in 5 ml PBS with 40 kHz for 3 min using an ultrasonic bath and preparing serial dilutions of this solution, which were plated on TSA plates for 24 h at 37°C.

### SDS page and western blot

*S. aureus* USA300 WT, Δpvl, or Δpvl+pvl strains were cultured as described above. The following day overnight cultures were normalized to the same optical density at 600 nm (OD_600_). Bacterial cells were pelleted by centrifugation at 3200 rcf for 10 min. Proteins in the supernatants were passed through a 0.2 μm-pore-size filter and precipitated with methanol-chloroform method at 4°C. Precipitated proteins were air-dried, resuspended with Lämmli + β-mercaptoethanol SDS loading buffer, and boiled for 5 min at 95°C. Proteins separated on 12% SDS-PAGE gels (Biorad, Mini-PROTEAN TGX Precast Gels, Basel, Switzerland) were transferred to 0.2 μm nitrocellulose membranes (Biorad, Trans-Blot Turbo), and probed sequentially with rabbit anti-*S. aureus* LukS-PV polyclonal antibody (IBT Bioservices, Rockville, USA) at 0.5 μg/ml. HPR-conjugated goat anti-rabbit IgG (Life Technologies, Basel, Switzerland) (1:10000) was used as a secondary antibody in a mixture with PBS supplemented with 0.1% Tween 20. Blots were developed using the Amersham ECL Prime Western Blotting Detection Reagent (Cytiva, Grens, Switzerland). The chemiluminescence signal was detected using the imaging system Syngene Chemi Genius (Cambridge, UK) and the GeneSys image acquisition software (GeneSnap Product version 7.12).

### HuBRGSF mice

#### Animal ethics statement

The animal studies were carried out in an Association for Assessment and Accreditation for Laboratory Animal Care (AAALAC) International accredited facility and were approved by the ethical committee of the canton of Graubünden in Switzerland (approval numbers 09_2021 and 17_2021).

#### Animals

Thirty specific and opportunistic pathogen-free (SOPF) female huBRGSF (genOway, Lyon, France), at 18 to 25 weeks of age were enrolled in this study. The huBRGSF were generated by transplanting newborn (≤ 5 days of age) mice intra-hepatically with ~0.9 × 10^5^ human hematopoietic progenitor cells (hHPC: CD34^+^ cord blood cells; Lonza, Morristown, NJ, USA), 24 h after full body irradiation conditioning (2.8 Gy; X-ray source). All mice were acclimatized for 2 weeks before surgical intervention, and mice were housed in individually ventilated cages (IVCs; Techniplast, Schwerzenbach, Switzerland and Allentown, Schlieren, Switzerland) with 2 to 6 mice per cage and under 12 h light/dark cycle. The cages were enriched with a plastic house, paper, and wood for gnawing and mice were provided *ad libitum* both water and food (#3436 for BALB/c mice and #3432 for huBRGSF; Provimi Kliba AG, Kaiseraugst, Switzerland).

The huBRGSF were operated in two batches. Therefore, four or six huBRGSF mice were allocated over three groups (inoculation with USA300 WT, Δpvl, or Δpvl+pvl) based on the humanization percentage of the mice (or the extent of human CD45+ cells assessed as described previously^(^^30^^)^). The average humanization percentage of the mice/group was evenly distributed for the three groups. Nine days before surgery and during the acclimatization period, the huBRGSF received intraperitoneally 10 μg human FMS-related Tyrosine Kinase 3 Ligand (Flt-3 L; Bio X Cell, Lebanon, NH, USA) diluted in sterile PBS as a treatment. This treatment was repeated on 7-, 5-, and 2-days pre-surgery. To assess the influence of host genetics, the aforementioned infection studies (USA300 WT, Δpvl, or Δpvl+pvl strains) were performed in WT BALB/c mice (*n* = 5/experimental group), the parental mouse strain from which the BRGSF were derived.

#### Anesthesia and analgesia

Before the surgical intervention, mice were anesthetized with sevoflurane (ca. 7% in O_2_, flow rate 1 l/min; Baxter AG, Opfikon, Switzerland), and sedation was maintained with sevoflurane (ca. 2–3% in O_2_, flow rate 0.6–0.8 l/min). Intraoperative analgesia consisted of 0.003 mg of buprenorphine given in a subcutaneous injection (Bupaq, Streuli Pharma AG, Switzerland) and post-operative analgesia was maintained with 0.2 mg/ml Tramal (Grünenthal Pharma, Mitlödi, Switzerland) in the drinking water during the entire post-operative period until euthanasia. Additional analgesia was given to decrease the burden resulting from the infection, which consisted of tramal (0.4 mg/ml in drinking water) and subcutaneous buprenorphine injections (twice daily 0.003 mg). Three animals (one in each group) had prolonged anesthesia due to post operative micro-CT analysis.

#### Surgical intervention

After placing the mouse in dorsal recumbency, the fur was clipped, and the surgery site was aseptically prepared. A hole was pre-drilled from the medial to lateral cortex of the proximal right tibia, 2–3 mm below the tibial plateau using a 25 G × 1″ needle as described previously.^(^^39^^)^ The pre-inoculated, L-shaped pin (see details above) was placed within this pre-drilled hole with the bent part of the pin secured under the skin.^(^^39^^)^ The wound was sutured using 5–0 vicryl rapide (Ethicon, Courcelles, Belgium).

#### Post-operative

Throughout the post-operative period, an animal welfare assessment was carried out twice daily by a veterinarian considering behavior, breathing, external appearance, urine, feces, mouse grimace scale, surgery site, and weight bearing. Each parameter was scored (0–3 or 0–4). The human endpoint was set at a score sum ≥9. Collectively, these clinical parameters were tabulated as clinical outcome scores, with the higher the score, the worse the outcome The weight of the mice was measured before the first Flt-3 L treatment, immediately before surgery, after the surgery, and before euthanasia. Euthanasia was performed under sevoflurane anesthesia by cervical dislocation.

Four mice per group (total *n* = 12) were processed for histology and received a pin inoculated with 5 × 10^5^ CFUs. One mouse inoculated with the USA300 Δpvl+pvl strain had to be excluded from the study as it was euthanized one day post-surgery because it met the humane endpoint. Final group sizes: *n* = 4 for the USA300 WT and USA300 Δpvl groups and *n* = 3 for the USA300 Δpvl+pvl group. Six mice per group (total *n* = 18) received a pin inoculated with 5 × 10^4^ CFUs and were processed for estimating the bacterial burden, enumeration of bone marrow immune cell populations by flow cytometry, human cytokine/chemokine measurements, and western blot.

After the mice were euthanized, one of the mice belonging to the USA300 Δpvl group had to be excluded because it had an abdominal cut (obtained during surgery preparation) that was inflamed. Group sizes: *n* = 6 for the USA300 WT group, *n* = 5 for the USA300 Δpvl group, and *n* = 6 for the USA300 Δpvl+pvl group.

#### CFU quantification

Soft tissue, tibial bones, and half of the organs (spleen, liver, kidney, and heart), cut lengthwise, were homogenized mechanically using an Omni Tissue Homogenizer and Hard Tissue Homogenizing tips (both Omni International, Kennesaw, GA, USA). Implants were sonicated with 40 kHz for 3 min in an ultrasonic bath. Soft tissue, bone, bone marrow, organ, and implant suspensions were serially diluted and 10 μl smears of these dilutions were plated in triplicate on 5% horse blood agar plates (Oxoid). Additionally, 200 μl of undiluted sample was spread onto 5% horse blood agar plates. Plates were incubated at 37°C, and the number of CFUs was determined after 48 h of incubation. Forthy-eight h values are indicated in the results.

#### Flow cytometric analysis

Single-cell suspensions of bone marrow (0.5 × 10^5^ cells) were obtained as described previously.^(^^5^^)^ In short, soft tissue was removed from the tibiae, the outer ends of the tibiae were removed, and the bone marrow cells were flushed out from the tibiae with a 24 G × 1″ needle attached to a 2 ml syringe containing Hanks’ buffered salt solution (HBSS; Gibco). The collected cells were passed through a 70 μm cell strainer and the red blood cells were removed by lysis. The obtained cells were then incubated with the Fixable Viability Dye eFluor™ 780 (Invitrogen, Schlieren, Switzerland) for 30 min at 4°C, washed with HBSS with 0.5% (w/v) fetal bovine serum (FBS; Sigma-Aldrich) and 2 mM ethylenediaminetetraacetic acid (EDTA; Sigma-Aldrich). Non-specific binding of antibodies to cells was blocked by incubation with human and mouse FC-receptor blocking reagents (Miltenyi Biotec, Solothurn, Switzerland or BD Bioscience, Allschwil, Switzerland, respectively) for 10 min at 4°C. Then, cells were stained with antibodies against myeloid markers (Table 1; all Biolegend, Fell, Germany) for 45 min at 4°C. Cells were then washed two times with HBSS with 0.5% (w/v) FBS and 2 mM EDTA, and acquired with the flow cytometer BD FACSAria III (BD Bioscience, Allschwil, Switzerland). See the supplement for the gating strategies applied within this study (Supplementary Figure 1 and 2A).

**Table 1:** A list of all antibodies used for flow analysis in this study.

Target	Antibody	Tag
Murine hematopoietic stem cells	anti-mouse CD45 antibody, #103114	PE/Cyanine 7
Human hematopoietic stem cells	anti-human CD45 antibody, #368526	BV510
Human dendritic cells	anti-human CD11c antibody, #337214	FITC
Human polymorphonuclear cells	anti-human CD66b antibody, #305106	PE
Human monocytes/MΦ	anti-human CD14 antibody, #325608	APC
Human NK cells	anti-human CD56 (NCAM) antibody, #318316	AF700
Human HLA-DR surface receptor	anti-human HLA-DR antibody, #307642	BV785

### Multiplex assay

Human proteins IL-8, TNFα, IL-6, IL-1β, IL-27, and IL-10 were quantiatedby using a U-plex, multiplex assays, Biomarker Group 1 (Human; MSD, Rockville, MD, United States) in serum samples and bone marrow aspirates of huBRGSF collected at day 3 post-surgery. Cytokines were also measured in supernatants obtained after centrifugation of isolated bone marrow cells in HBSS. Bone marrow samples were concentrated (~10x) using the Amicon Ultra-0.5 centrifugal filter unit with 3KDa MWCO (Merck).

### Histology

#### Histochemical stains

The fixed right tibiae were rinsed in MilliQ water and placed into a decalcifying solution containing 12.5% (w/v) EDTA(Roth AG, Arlesheim, Switzerland) and 1.25% (w/v) sodium hydroxide (Sigma-Aldrich) for 6 to 8 days. Thereafter, samples were dehydrated with an ascending ethanol gradient. Pins were removed, and the tibial bone with soft tissue were embedded in paraffin.

#### Histological stains

5 μm tissue sections were mounted on glass slides, deparaffinized, and rehydrated. Sections were stained with hematoxylin & eosin (H&E) or Brown and Brenn (BB) as described previously.^(^^39^^,^^40^^)^ Images were taken with a VS120 Virtual Slide Microscope (Olympus, Waltham, MA, USA). Higher magnification (40X) images were acquired with Olympus BX40 light microscope.

#### Immunofluorescent stains

The following primary antibodies were used for immunofluorescent staining of HuBRGSF infected limbs (USA300 WT, USA300 Δpvl, or USA300 Δpvl+pvl): mouse anti-CEACAM8/CD66b for neutrophils (Clone G10F5, NBP2–80664, Novus Biologicals, Zug, Switzerland), rabbit anti-*S. aureus* (PA1–7246, RRID:AB_561546, Thermo Fisher Scientific), and mouse anti-CD68 for monocytes (Clone PG-M1, MS-1808-S1, RRID:AB_149350, Thermo Fisher Scientific). Primary antibodies were used at 1:50 dilution.

Primary antibodies were visualized by incubation with the following secondary antibodies all from Jackson ImmunoResearch Laboratories (PA, USA): Cy3-conjugated goat anti-mouse IgM (115–165-020, RRID:AB_2338683), Alexa Fluor 488-conjugated donkey anti-rabbit IgG (711–546-152, RRID:AB_2340619), Alexa Fluor 647-conjugated donkey anti-mouse IgG (715–606-150, RRID:AB_2340865). Slides were incubated with secondary antibodies and streptavidin at 1:200 dilution.

Immunofluorescent staining was performed as described previously.^(^^30^^)^ Briefly, 5 μm paraffin bone sections were incubated at 60°C for deparaffinization. Tissue sections were transferred to xylenes and gradually hydrated by sequentially transferring into alcohol, 95% alcohol, 70% alcohol, and water. Sections were then immersed in antigen retrieval solution (S1699, DAKOCYTOMATION, Santa Clara, CA, USA) and boiled for 30 min. Non-specific binding was blocked by incubating tissue sections with 5% normal donkey serum (Jackson ImmunoResearch Laboratories) at room temperature (RT) for 30 min in a humid chamber. Immediately after removing the blocking solution, primary antibodies were added to the tissue sections. Slides were incubated overnight with the primary antibodies at RT. Tissue sections were washed with PBS and secondary antibodies were incubated for 1 h at RT. Finally, tissues were washed with PBS and mounted with Vectashield antifade mounting media with DAPI (H-1200, Vector Laboratories, Newark, CA, USA). Images were taken with a Zeiss Axioplan 2 microscope (Jena, Germany) and collected with a Hamamatsu camera (Bridgewater, NJ, USA). *S. aureus* pictures were pseudo-colored green with software of Zeiss microscope.

### Data analysis

Kaluza Analysis Software (Beckman Coulter Life Sciences, Indianapolis, IN, USA) was used to evaluate flow cytometric data. GraphPad Prism 8 (GraphPad Software, San Diego, CA, USA) was used for statistical analysis of data and for data visualization. A Shapiro–Wilk test was performed to assess the normality of the data together with visually assessing Q-Q plots of the data. Parametric data were analyzed with a Tukey’s multiple comparison test part of a one-way or a two-way ANOVA, respectively, whereas non-parametric data were examined with a Dunn’s multiple comparison test part of a Kruskal-Wallis test. *P*-values of <0.05 were considered statistically significant.

## Results

### PVL is dispensable for in vitro planktonic, biofilm growth, coating of implants, and staphylococcal abscess community formation

**Figure 1:**
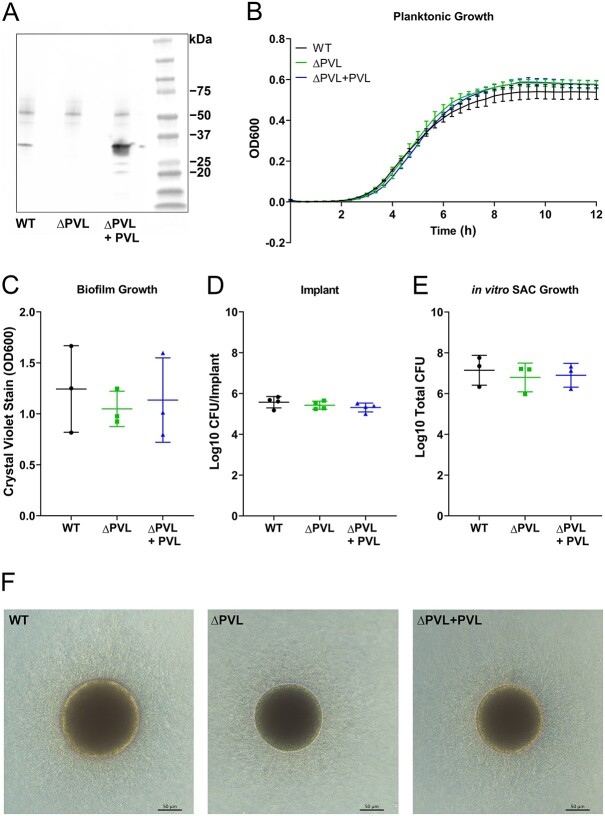
Deletion of PVL from the USA300 strain does not affect in vitro planktonic, biofilm, or staphylococcal abscess community (SAC) growth or its ability to coat stainless-steel implants. (A) Detection of the PVL subunit S (LukS-PV) at ~33 kDa by SDS page and western blotting in supernatants of USA300 WT (left), USA300 Δpvl (middle), or USA300 Δpvl+pvl (right). (B) Planktonic exponential and stationary phase growth of USA300 WT (circle), USA300 Δpvl (square), and USA300 Δpvl+pvl (triangle) measured over a 12 h time period. (C) Biofilm quantification by measuring crystal violet stains of USA300 WT, USA300 Δpvl, or USA300 Δpvl+pvl biofilm. (D) CFUs retrieved from stainless-steel implants after being coated with USA300 WT, USA300 Δpvl, or USA300 Δpvl+pvl. (E) CFU quantification (per SAC sample) and (F) morphological appearance of SACs from USA300 WT, USA300 Δpvl, or USA300 Δpvl+pvl. Data (B-E) are mean ± SD and from 3 or 4 independent experiments.

Using immunoblot analyses, we first confirmed that the USA300 WT strain and the complemented USA300 Δpvl+pvl secrete PVL (LukS-PV detection ~33 kDa), whereas the mutant USA300 Δpvl does not (Figure 1A). Growth patterns of the strains were checked to ensure that the USA300 WT, Δpvl, and Δpvl+pvl strains grow in a similar manner. When the strains were grown planktonically over a 12 h period, the strains USA300 WT, Δpvl, and Δpvl+pvl reached stationary phase after approximately 9 h, and no differences were observed in planktonic growth between the three strains (Figure 1B). Biofilm growth (Figure 1C), attachment to stainless-steel implants (Figure 1D), and in vitro SAC growth (Figure 1E) were similar for the three strains, and no morphological differences were observed in the in vitro SACs (Figure 1F).

### Improved clinical outcomes for huBRGSF infected with the isogenic PVL mutant MRSA strain

**Figure 2:**
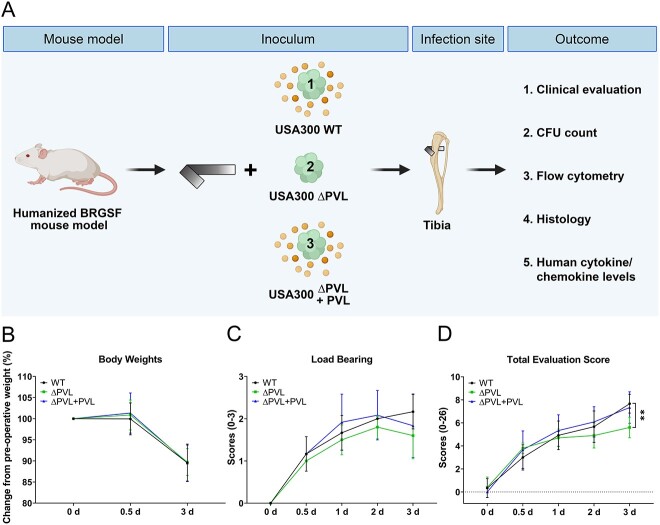
PVL worsens clinical outcomes in huBRGSF at 3 d post-surgery (A) overview of the in vivo study design. HuBRGSF were infected with either USA300 WT, USA300 Δpvl, or USA300 Δpvl+pvl by placing a pre-inoculated pin trans-cortically in the proximal part of the tibia, which after 3 d resulted in a transtibial implant-associated osteomyelitis. Clinical evaluation included (B) body weight loss compared to the pre-operative weight (set to a 100%), (C) load bearing on the operated, right leg scores ranging from 0–3, and (D) total clinical evaluation scores ranging from 0–20 of mice infected with WT (circle), Δpvl (square), or Δpvl+pvl (triangle), which were determined at 0, 0.5, 2, and 3 d post-operative. Body weight loss data are medians with 95% confidence intervals. Load bearing and total evaluation scores are mean ± SD. Data was analyzed with a Tukey’s multiple comparison test of a two-way ANOVA. *N* = 6 for WT-infected mice, *n* = 5 for Δpvl-infected mice, and *n* = 6 for Δpvl+pvl-infected mice. ^*^^*^p < 0.01.

The importance of PVL during human osteomyelitis remains unknown. Hence, we utilized a well-validated humanized mouse implant-associated osteomyelitis model^(^^8^^,^^30^^,^^39^^,^^41–43^^)^ to assess PVL virulence during infection (Figure 2A). Mice were separated into three groups according to the strain used for the inoculum. Humanization rates of mice belonging to these three different groups were similar at the start of the study: 79 ± 8.24% for the USA300 WT group, 80.15 ± 3.86% for the USA300 Δpvl group, and 80.3 ± 3.81% for the USA300 Δpvl+pvl group (Supplementary figure 2B). WT-, Δpvl-, or Δpvl+pvl-infected huBRGSF mice had comparable weight losses after surgery (Figure 2B) and load bearing on the operated right leg (Figure 2C). However, compared to Δpvl-infected huBRGSF, at 3 days post-surgery, WT-infected huBRGSF mice had significantly higher total clinical evaluation scores (*p* = 0.0096; Figure 2D), and Δpvl+pvl-infected huBRGSF also had higher total clinical evaluation scores (Figure 2D). Three days post-infection, humanization rates in bone marrow were variable for Δpvl- (60.66 ± 30.81%) or Δpvl+pvl- (66.97 ± 19.10%) infected mice and was 84.4 ± 11.69% for the WT group (Supplementary figure 2D).

### HuBRGSF mice infected with isogenic PVL mutant had less severe bone infection and sepsis

**Figure 3:**
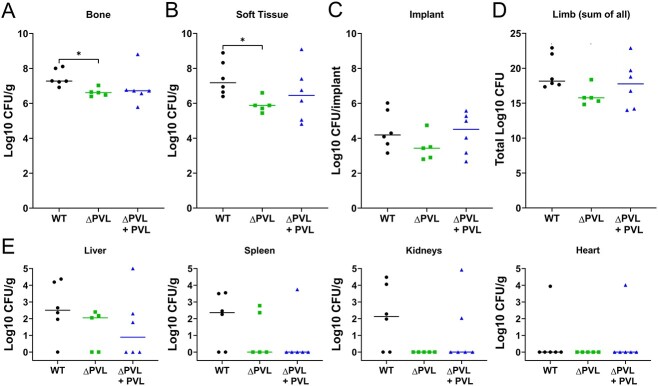
Isogenic PVL mutant induced a less severe bone infection in huBRGSF mice. Bacterial load of (A) bone, (B) soft tissue, and (C) implants from WT-, Δpvl-, or Δpvl+pvl-infected huBRGSF at 3 d post-infection, and (D) the cumulative CFUs per limb from mice of the three tested groups. (E) Bacterial load in liver, spleen, kidney, and/or heart tissue of WT- (circle), Δpvl- (square), or Δpvl+pvl-- (triangle) infected mice at 3 d post-infection. CFU data are log_10_ transformed medians and was analyzed with a non-parametric Kruskall-Wallis one-way ANOVA in combination with a Dunn’s multiple comparison test. *N* = 6 for WT-infected mice, *n* = 5 for Δpvl-infected mice, and *n* = 6 for Δpvl+pvl-infected mice. ^*^p < 0.05.

Previous studies showed that *S. aureus* pathogenesis during pneumonia and SSTI depends on PVL.^(^^28^^,^^29^^)^ To assess whether PVL is also required for *S. aureus* virulence during acute implant-associated osteomyelitis, the bacterial load of the operated, right leg consisting of soft tissue, the implant, and bone of WT-, Δpvl-, or Δpvl+pvl-infected huBRGSF was evaluated at 3 days post-surgery. Bone of WT-infected huBRGSF mice had a significantly higher bacterial load than Δpvl-infected huBRGSF mice (7.47 Log_10_ CFU *vs*. 6.63 Log_10_ CFU, *p* = 0.0189; Figure 3A). Also, the soft tissue of WT-infected huBRGSF contained significantly more bacteria than Δpvl-infected huBRGSF (7.43 Log_10_ CFU *vs*. 5.90 Log_10_ CFU, *p* = 0.0403; Figure 3B). No differences were noted for the bacteria retrieved from the implant of huBRGSF from the three tested groups (Figure 3C). The sum of bacterial load of bone, soft tissue, and the implant showed that the limbs of WT-infected huBRGSF mice contained more CFUs than limbs of Δpvl-infected huBRGSF mice (19.38 Log_10_ CFU *vs*. 16.0 Log_10_ CFU; Figure 3D). No correlations were found between the percentage of human CD45^+^ cells in blood pre-operatively and the number of CFUs detected in tibial bone of huBRGSF (Supplementary figure 2D). Interestingly, the percentage of human CD45^+^ cells in bone marrow, 3 days post-operatively, correlated negatively with the number of CFUs detected in bone of only WT-infected huBRGSF (*p* = 0.0028; Supplementary figure 2E).

Additionally, the presence of bacteria in the internal organs (liver, spleen, kidneys, and heart) was assessed as a measure of systemic dissemination. WT-infected huBRGSF had the most culture positive internal organs, with 14 organs out of 24 organs. In contrast, Δpvl-infected huBRGSF had 6 out of 20 organs culture positive. Δpvl+pvl-infected huBRGSF had 7 culture positive organs out of 24 organs (Figure 3E). Overall, the lack of PVL made *S. aureus* less virulent during acute implant-associated osteomyelitis in huBRGSF, as Δpvl-infected huBRGSF had fewer CFUs within bone and soft tissue of the inoculated limb and less culture-positive organs.

### Isogenic PVL mutant does not induce a less severe bone infection phenotype in BALB/c mice

To evaluate if the observed phenotype in huBRGSF mice was primarily facilitated by their functional human myeloid cells, we performed an in vivo study with BALB/c mice infected with USA300 WT, Δpvl, or Δpvl+pvl. The differences observed in bacterial load between WT- and Δpvl-infected huBRGSF mice were not detected in WT- and Δpvl-infected BALB/c mice (Supplementary figure 3A-E). However, Δpvl+pvl-infected BALB/c mice did have a significantly lower number of bacteria in their bone (6.12 Log_10_ CFU *vs.* 4.49 Log_10_ CFU, *p* = 0.0155), but a higher bacterial load in their spleen (0.47 Log_10_ CFU *vs*. 2.14 Log_10_ CFU, *p* = 0.0272) than WT-infected BALB/c mice. Nonetheless, these results indicate that human myeloid cells are most likely required for *S. aureus* pathogenesis in huBRGSF mice.

### Isogenic PVL mutant had impaired accumulation of human myeloid cells and cell death in the huBRGSF bone niche

**Figure 4:**
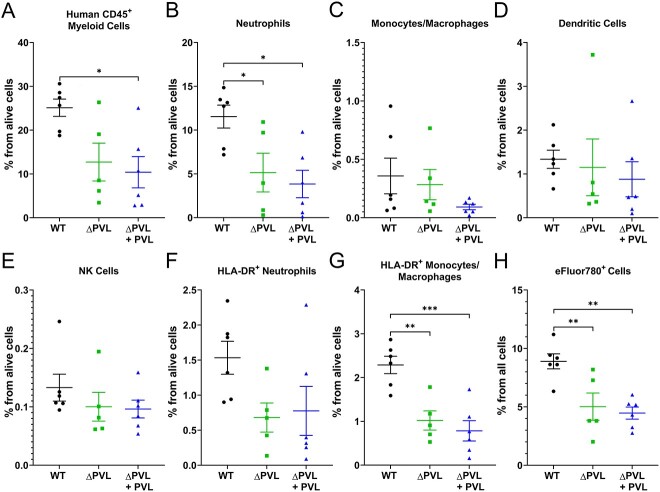
HuBRGSF mice infected with isogenic PVL mutant had fewer human myeloid cells and dead cells in the bone niche. Myeloid cell populations in bone marrow of huBRGSF mice with a transtibial implant-associated osteomyelitis after infection with MRSA USA300 WT, USA300 Δpvl, or USA300 Δpvl+pvl. Percentages of (A) human CD45^+^ myeloid cells, (B) neutrophils, (C) monocytes/macrophages, (D) dendritic cells, (E) NK cells, (F) HLA-DR^+^ neutrophils, (G) HLA-DR^+^ monocytes/macrophages, or (H) eFluor780^+^ dead cells in bone marrow of WT- (circle), Δpvl- (square), or Δpvl+pvl- (triangle) infected mice, 3 d after surgery are depicted. Data are mean ± SD and was analyzed with a Tukey’s multiple comparison test of a one-way ANOVA. *N* = 6 for WT-infected mice, *n* = 5 for Δpvl-infected mice, and *n* = 6 for Δpvl+pvl-infected mice. ^*^p < 0.05, ^*^^*^p < 0.01, ^*^^*^^*^p < 0.001.

PVL lyses human neutrophils, monocytes, and macrophages.^(^^15^^)^ In a humanized NSG mice pneumonia model, macrophages were significantly increased in the bronchoalveolar lavage fluid (BALF) of mice infected with a strain of *S. aureus* lacking PVL.^(^^28^^)^ Here, we examined human myeloid cells in the bone marrow of WT-, Δpvl-, or Δpvl+pvl-infected huBRGSF with flow cytometry to determine the impact of PVL on these cells during acute osteomyelitis. WT-infected mice had higher percentages of human CD45^+^ myeloid cells within their bone marrow than Δpvl or Δpvl+pvl-infected huBRGSF (*p* = 0.0522 and *p* = 0.0157, respectively; Figure 4A). The increase in CD45+ hematopoietic cells is likely associated with the significant increase in bone marrow neutrophils in the WT-infected huBRGSF compared to Δpvl or Δpvl+pvl-infected huBRGSF mice (*p* = 0.0471 and *p* = 0.0125, respectively; Figure 4B). No differences were detected in monocyte/macrophage, dendritic cell, NK cell, and HLA-DR^+^ neutrophil percentages between the experimental groups (Figure 4C-F). However, the bone marrow of WT-infected huBRGSF mice contained significantly higher percentages of HLA-DR^+^ monocytes/macrophages than Δpvl or Δpvl+pvl-infected huBRGSF mice (*p* = 0.0033 and *p* = 0.0005, respectively; Figure 4G). The percentage of eFluor780^+^ dead cells from all bone marrow cells was also higher in WT-infected huBRGSF compared to Δpvl or Δpvl+pvl-infected animals (*p* = 0.0097 and *p* = 0.0026, respectively; Figure 4H). It appeared that *S. aureus* lacking PVL had fewer myeloid cells infiltrating the infection site, and fewer cells were apoptotic.

### Isogenic PVL mutant does not form SACs in bone marrow of huBRGSF mice

**Figure 5:**
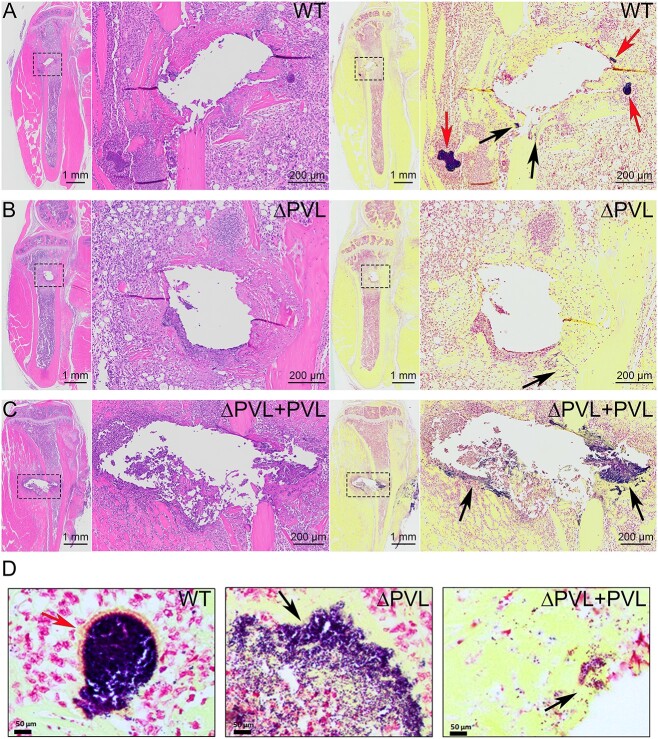
The isogenic PVL mutant does not form staphylococcal abscess communities (SACs) in bone marrow of huBRGSF mice. Histological evaluation of hematoxylin and eosin stained (left panel) or Brown and Brenn (BB) stained (right panel) tibiae of huBRGSF mice 3 d post-inoculation with either MRSA (A) USA300 WT, (B) USA300 Δpvl, or (C) USA300 Δpvl+pvl. (D) Higher magnification images (40X) of bacteria positive areas of BB stained sections. Black arrows indicate areas that stained positive for bacteria but that were not SAC structures, whereas red arrows point to SACs.

Humanized NSG mice develop more SACs than WT C57BL/6 mice during *S. aureus* implant-associated osteomyelitis.^(^^30^^)^ Moreover, a recent publication highlighted that lysis of human neutrophils by PVL leads to thrombus generation and additional fibrin formation,^(^^44^^)^ which are essential building blocks for generating SACs. Therefore, we hypothesized that SAC formation is PVL-dependent. Histopathological assessment of SAC formation revealed that tibiae of WT-infected huBRGSF contained bacterial aggregates and SAC structures (with pseudocapsules) at the location near the inoculated pin (black or red arrows, respectively; Figure 5A and D), whereas tibiae of Δpvl-infected huBRGSF only contained areas with scattered small aggregates of bacteria (black arrow; Figure 5B and D), but no SACs. Tibiae of Δpvl+pvl-infected huBRGSF seemingly contained large areas that stained positive for bacteria (black arrows; Figure 5C and D), but lacked the features of a classic SAC structure. *S. aureus*-specific immunostaining confirmed our observations (Supplementary Figure 4A-C). We noticed that the bone marrow of WT-infected huBRGSF contained *S. aureus* SACs, whereas bone marrow of Δpvl- or Δpvl+pvl-infected huBRGSF mice lacked SACs (green; Supplementary figure 4A-C).

### Minor differences in human cytokine levels of huBRGSF infected with the isogenic PVL mutant strain

**Figure 6:**
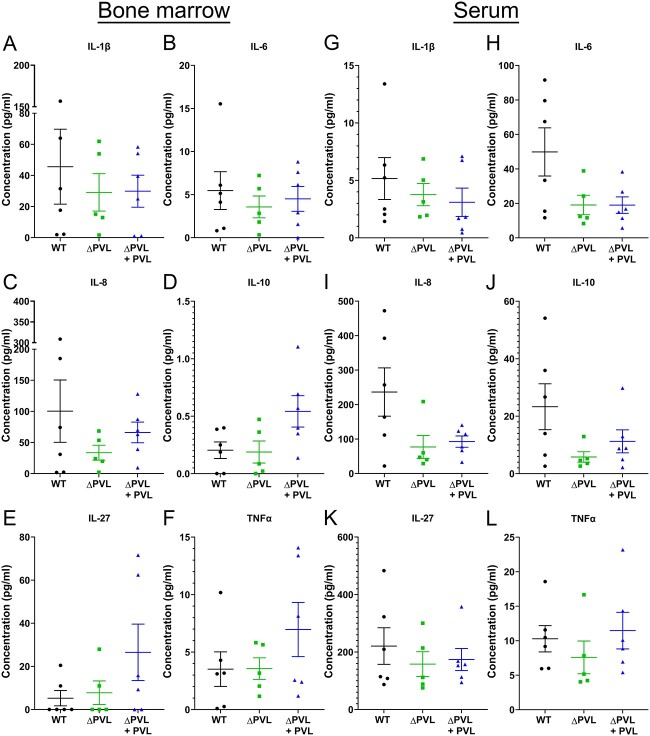
Minor differences in human serum cytokine levels in huBRGSF mice infected with the isogenic PVL USA300. Human cytokine concentrations in huBRGSF mice with a transtibial implant-associated osteomyelitis due to MRSA USA300 WT, USA300 Δpvl, or USA300 Δpvl+pvl. Human IL-1β, IL-6, IL-8, IL-10, IL-27, and TNFα concentrations were evaluated within (A-F) bone marrow homogenates or (G-L) serum of WT- (circle), Δpvl- (square), or Δpvl+pvl- (triangle) infected mice 3 d after surgery. Data are mean ± SD, and *n* = 6 for WT-infected mice, *n* = 5 for Δpvl-infected mice, and *n* = 6 for Δpvl+pvl-infected mice.

To further characterize systemic immunity, we measured human cytokines IL-1β, IL-6, IL-8, IL-10, IL-27, and TNFα in bone marrow homogenates and serum of the *S. aureus* infected huBRGSF mice (Figure 6). Only minor differences in the concentration of human cytokines/chemokines were observed in the bone marrow homogenate samples of the WT-, Δpvl-, or Δpvl+pvl-infected huBRGSF mice(Figure 6A-F). Two Δpvl-infected huBRGSF mice had higher human IL-6, IL-8, and IL-1β concentrations in their bone marrow, which corresponded to more human myeloid cells, neutrophils, HLA-DR+ monocytes/macrophages and fewer murine myeloid cells shown above in Fig. 4. WT-infected huBRGSF had a higher concentration of human IL-6 and IL-8 in their serum compared to Δpvl-infected huBRGSF mice, while the other cytokine concentrations were not different between the three groups (Figure 6G-L).

## Discussion

In this study, we showed for the first time that PVL contributes to *S. aureus* pathogenicity during acute implant-associated osteomyelitis in a novel humanized BRGSF mouse model with functional human myeloid cells. We proved that huBRGSF infected with an isogenic USA300 mutant lacking PVL had significantly better clinical outcomes, reduced bacterial load in peripheral organs, bone, and soft tissue, and no SAC formation in bone marrow compared to WT-infected animals. Importantly, these animals had significant alterations in the infiltration by human myeloid cells and cell death. Besides the previously used humanized non-obese diabetic (NOD)–scid IL2Rγnull (NSG) mouse model,^(^^28–30^^,^^45^^)^ here, we demonstrated that humanized BRGSF with functional human neutrophils and monocytes/macrophages are a viable rodent model to study human-specific *S. aureus* pathophysiology in vivo.

Previous studies with humanized NSG mice evaluated the impact of PVL in *S. aureus* in pneumonia^(^^28^^)^ and SSTI infections.^(^^29^^)^ Similar to our results, the humanized NSG mice infected with an *S. aureus* Δpvl mutant had less severe disease compared to WT *S. aureus* infected animals with smaller skin lesions during SSTI^(^^29^^)^ and fewer bacteria in lung tissue and BALF^(^^28^^)^ during lung infections. In contrast to our results, 24 hours after inoculation, the Δpvl *S. aureus* infected NSG mice also had more human macrophages and murine neutrophils and higher concentrations of human TNFα and IL-6 in their BALF than WT *S. aureus* infected NSG mice.^(^^28^^)^ We observed the opposite; WT-infected huBRGSF had more innate immune cells in bone marrow than Δpvl-infected huBRGSF mice at 3 days post-inoculation. It may be that during the first 24 h post-inoculum there was a drop in the number of innate immune cells in bone marrow of WT-infected huBRGSF, but as time progressed and *S. aureus* numbers increased, a larger influx of innate immune cells to the infection site was initiated, likely to compensate for the loss of lysed innate cells. Our observations were performed 3 days post inoculation, unlike the previous study where samples were harvested 1 day after infection.^(^^28^^)^ Additionally, the genetic Th2 type BALB/c background could be contributing to the differences in innate immune cells observed in the NSG mice.^(^^46^^)^

While typical SAC structures were identified in the bone marrow of WT-infected huBRGSF, no such SACs were detected in the bone marrow of Δpvl-infected huBRGSF mice. Thus, the impact of PVL on human innate immune cells is essential for SAC formation during osteomyelitis. Additionally, cell debris generated by PVL lysis activity could contribute to *S. aureus* aggregation during abscess formation. Indeed, PVL has been detected in human bone abscesses previously.^(^^47^^)^ Additionally, others have shown that lysis of human neutrophils by PVL causes platelet activation and aggregation, which might subsequently lead to further thrombin and fibrin formation.^(^^44^^)^ Curiously, USA300 Δpvl+pvl infected huBRGSF did not have SACs in their bone marrow but large areas with planktonic *S. aureus* growth. The ability of PVL to lyse or activate human neutrophils, which are essential for SAC formation, is concentration-dependent.^(^^48^^)^ It is possible that higher PVL concentrations in their bone marrow may not be optimal for SAC formation. Additional studies are required to comprehensively examine this phenomenon.

Although we observed apparent differences in the numbers of innate immune cells in bone marrow of WT- or Δpvl-infected huBRGSF, surprisingly, there were no differences in human cytokine or chemokine concentrations in bone marrow homogenates between these two groups. Compared to the other mice of the same group, two Δpvl-infected huBRGSF mice had higher human IL-6, IL-8, and IL-1β concentrations in their bone marrow, which coincided with more human myeloid cells, neutrophils, HLA-DR^+^ monocytes/macrophages and less murine myeloid cells. PVL is known to bind to other *S. aureus* leukotoxins such as LukED, hlgAB, and HlgCB to form a non-cognate pairs with these leukotoxins.^(^^14^^)^ It may be that in the Δpvl-infected huBRGSF, one of these three leukotoxins becomes dominant and acquires a compensatory role because of PVL depletion. Instead, human cytokines and chemokines levels in WT-infected huBRGSF were overall higher compared to the other groups, which is potentially a result of having more *S. aureus* dissemination through the bloodstream to other organs than Δpvl-infected huBRGSF.

We evaluated the effect of PVL during the acute phase of osteomyelitis in huBRGSF but not during chronic osteomyelitis. For soft skin infections in rabbits, the influence of PVL during an acute *vs.* a chronic infection has been examined.^(^^49^^,^^50^^)^ Skin lesions in rabbits infected subcutaneously with a USA300 Δpvl strain were significantly smaller compared to lesions induced by PVL secreting USA300 strain, at 2 to 5 days post-infection.^(^^50^^)^ However, 6 days post-infection, skin lesions^(^^50^^)^ and abscess volume^(^^49^^)^ did not differ between WT- or Δpvl-infected rabbits. This suggests that PVL might not necessarily influence the course or severity of chronic infections, potentially because more organized *S. aureus* structures secrete less PVL than planktonic *S. aureus.*^(^^51^^)^ It would be interesting to investigate whether the reported differences between WT-infected huBRGSF and Δpvl-infected huBRGSF persist during the chronic osteomyelitis and potentially influence bone cells and the influx of adaptive immune cells. One study with a rabbit osteomyelitis model does show that rabbits challenged with PVL-negative *S. aureus* still had less infected bones, less bone deformation, and less muscle and joint involvement than rabbits inoculated with a PVL-positive *S. aureus* 28 days post-infection.^(^^52^^)^

The Δpvl+pvl-infected BALB/c mice had significantly fewer CFUs in bone tissue of the operated limb, while having more CFUs in their peripheral organs than WT-infected BALB/c mice. Although PVL has no reactivity towards murine cells, it has been suggested that PVL might be needed to stimulate host immune cell responses required for eradication of the bacterium.^(^^23^^)^ For example, PVL was able to modulate the secretion levels of cytokines and chemokines by murine neutrophils,^(^^27^^)^ and the depletion of PVL resulted in a significantly larger abscess area in BALB/cAnNHsd mice (SSTI model).^(^^23^^)^ Potentially, the clearance of bacteria from bone tissue is more effective due to high concentrations of PVL in Δpvl+pvl-infected BALB/c mice. Another possibility is that a different *S. aureus* virulence factor gives a phenotype within the Δpvl+pvl-infected BALB/c mice. It has been reported that upon expression of the *luk-PV* operon, secretion of Serine-Aspartate Repeat protein D (SdrD) and staphylococcal protein A (SpA) was upregulated.^(^^53^^)^ SpA is known for its interference with humoral responses and phagocytosis by immune cells,^(^^54^^)^ but once phagocyted it might also promote intracellular survival,^(^^55^^)^ potentially leading to dissemination into organ tissue^(^^56^^)^ by using these cells as “Trojan Horses”.^(^^6^^,^^43^^)^ We indeed observed higher CFUs in organs of Δpvl+pvl-infected BALB/c mice than WT- or Δpvl-infected BALB/c mice. Furthermore, we did not observe worsening of the implant-associated osteomyelitis in Δpvl-infected BALB/c mice, which is in line with other reports that used murine bacteremia, SSTI, and pneumonia models.^(^^23^^,^^25^^)^

A limitation of the study is that we did not use BRGSF mice engrafted with murine bone marrow cells exposed to a sublethal γ-irradiation-induced myeloablation as a control group. This would have made it possible to exclude any possible effects of the radiation procedure on infection development. It has been reported that BALB/c mice and NSG mice engrafted with BALB/c bone marrow cells had similar SSTIs with comparable bacterial loads when inoculated with 10^6, 107, and 108^ CFU.^(^^29^^)^ Also, NSG mice engrafted with cells of C57BL/6 J mice had a similar pulmonary infection as wild-type C57BL/6 J mice.^(^^28^^)^ Thus, it appears that the impact of radiation on at least the development of an infection in the lungs and skin and soft tissue is neglectable. Whether this holds true for bone infections should be explored in the future. Another limitation of the study is that the USA300 Δpvl+pvl strain did not fully recover the PVL knockout strain. A study with a rabbit SSTI model that used the same complemented USA300 Δpvl+pvl strain (LUG1515) also observed partial rescue of the PVL phenotype at the acute phase of infection.^(^^50^^)^ The authors concluded that this may be due to differences in the amount of PVL produced by either the wild-type strain or the complemented strain.^(^^50^^)^ We attempted to validate this for our study, but unfortunately, ex vivo CFUs in the bone tissue of the huBRGSF mice were too low to detect secreted PVL in the bone marrow homogenate samples with western blot. Lastly, we did not assess for the Δpvl+pvl-infected mice plasmid retention at the end of the study. This should be part of future studies to rule out that the partial recovery of the PVL phenotype in Δpvl+pvl-infected mice might be due to loss of the plasmid.

Overall, this study showed for the first time that the human-specific leukotoxin PVL contributes to *S. aureus* virulence during acute implant-associated osteomyelitis. Additionally, the use of the humanized mouse model allows to study immunopathogenesis, in the context of *S. aureus* infections and might be used to contribute to predicting anti-staphylococcal vaccine efficacy or therapy responsiveness.

## Supplementary Material

Sup_Figure_1_2_ziad005

Sup_Figure_2_2_ziad005

Sup_Figure_3_Revised_2_ziad005

Sup_Figure_4_2_ziad005

## Data Availability

Data of the current study are available from the corresponding author on reasonable request.
