# Supplementary figures and images for: Staphylococcus aureus Panton-Valentine Leukocidin worsens acute implant-associated osteomyelitis in humanized BRGSF mice
Source: JBMR Plus. 2024 Jan 4;8(2):ziad005. doi: 10.1093/jbmrpl/ziad005 (PMC10945728; doi:10.1093/jbmrpl/ziad005)

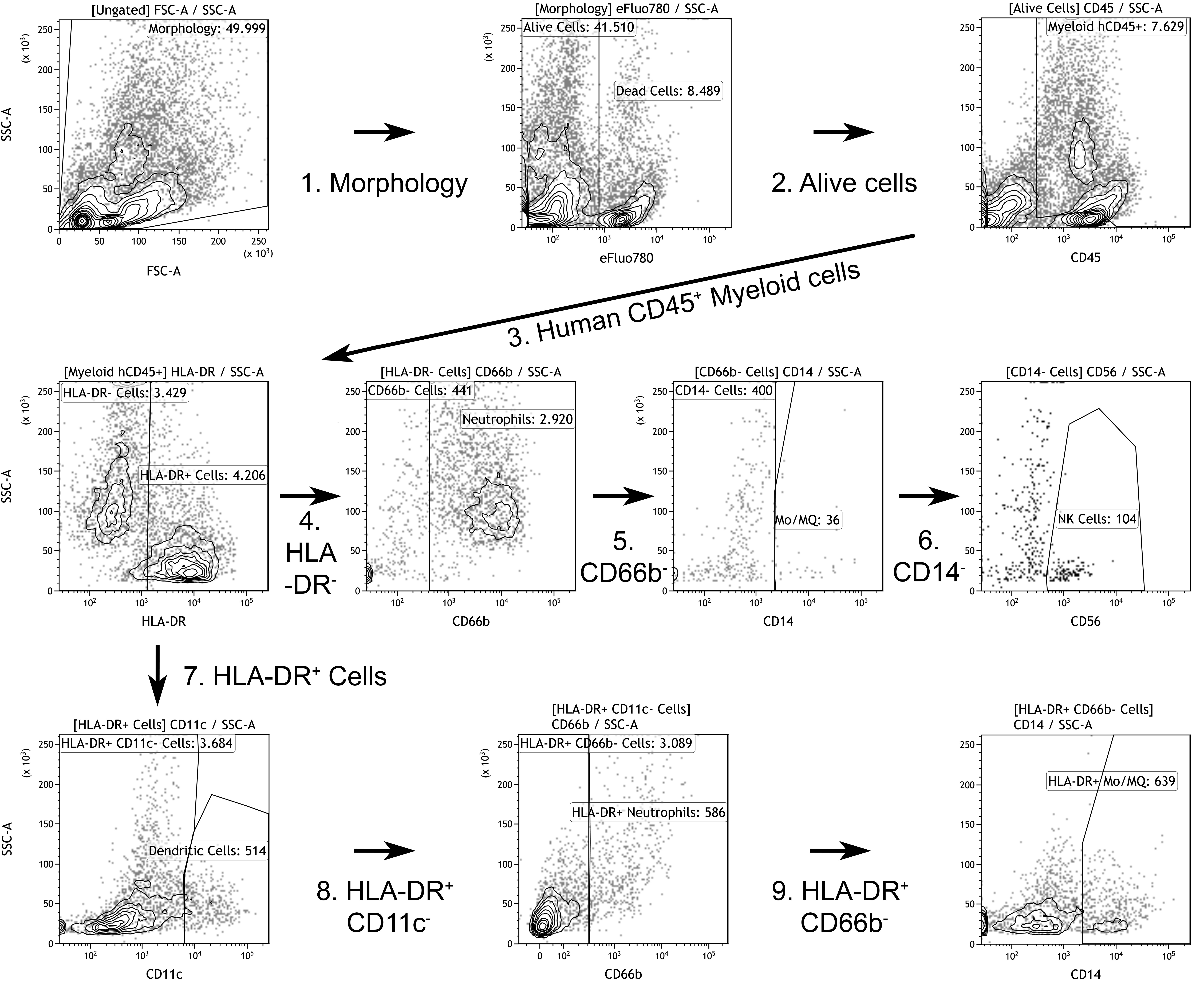

Supplement: Sup_Figure_1_2_ziad005 [file sup_figure_1_2_ziad005.jpeg]

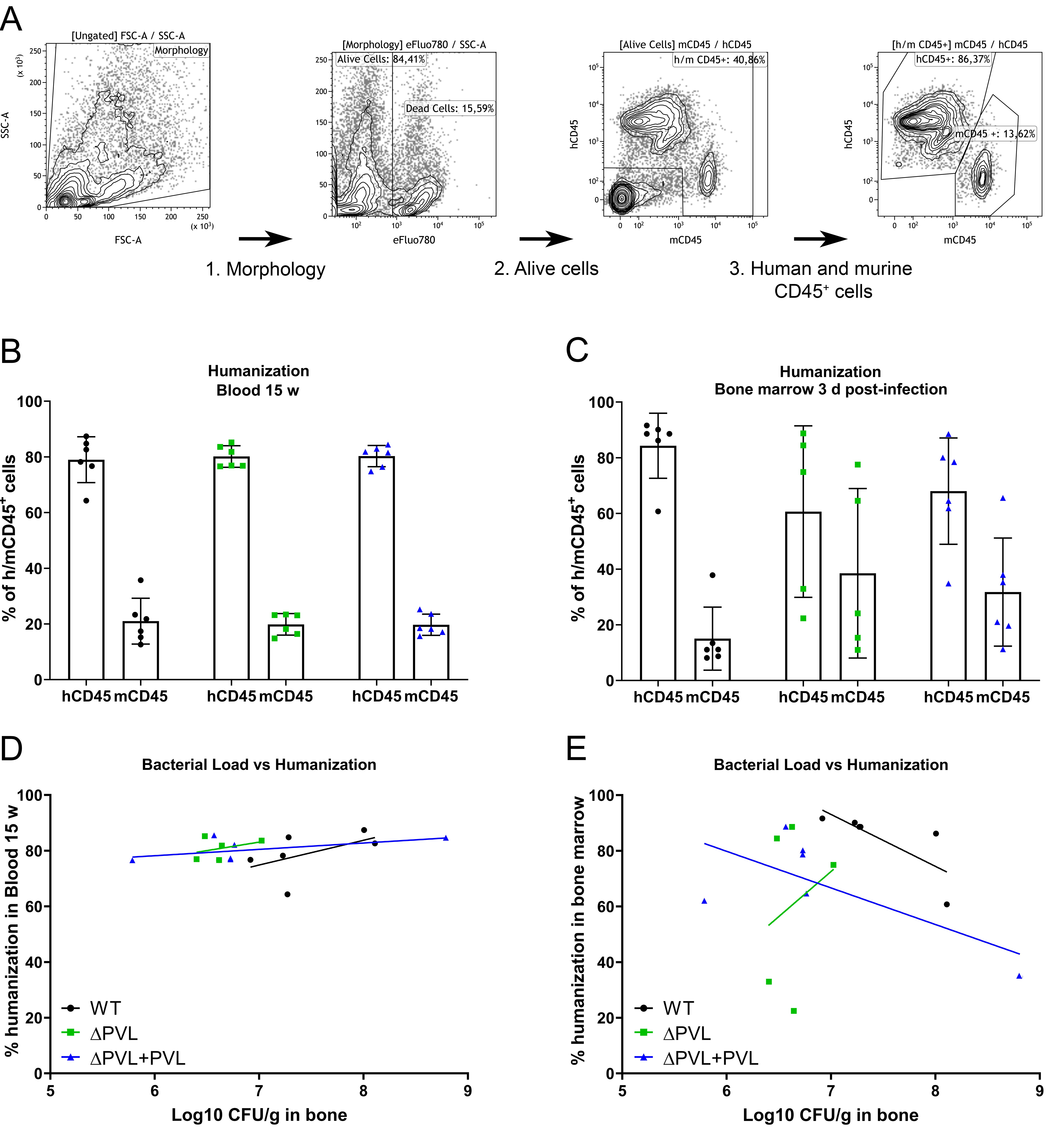

Supplement: Sup_Figure_2_2_ziad005 [file sup_figure_2_2_ziad005.jpeg]

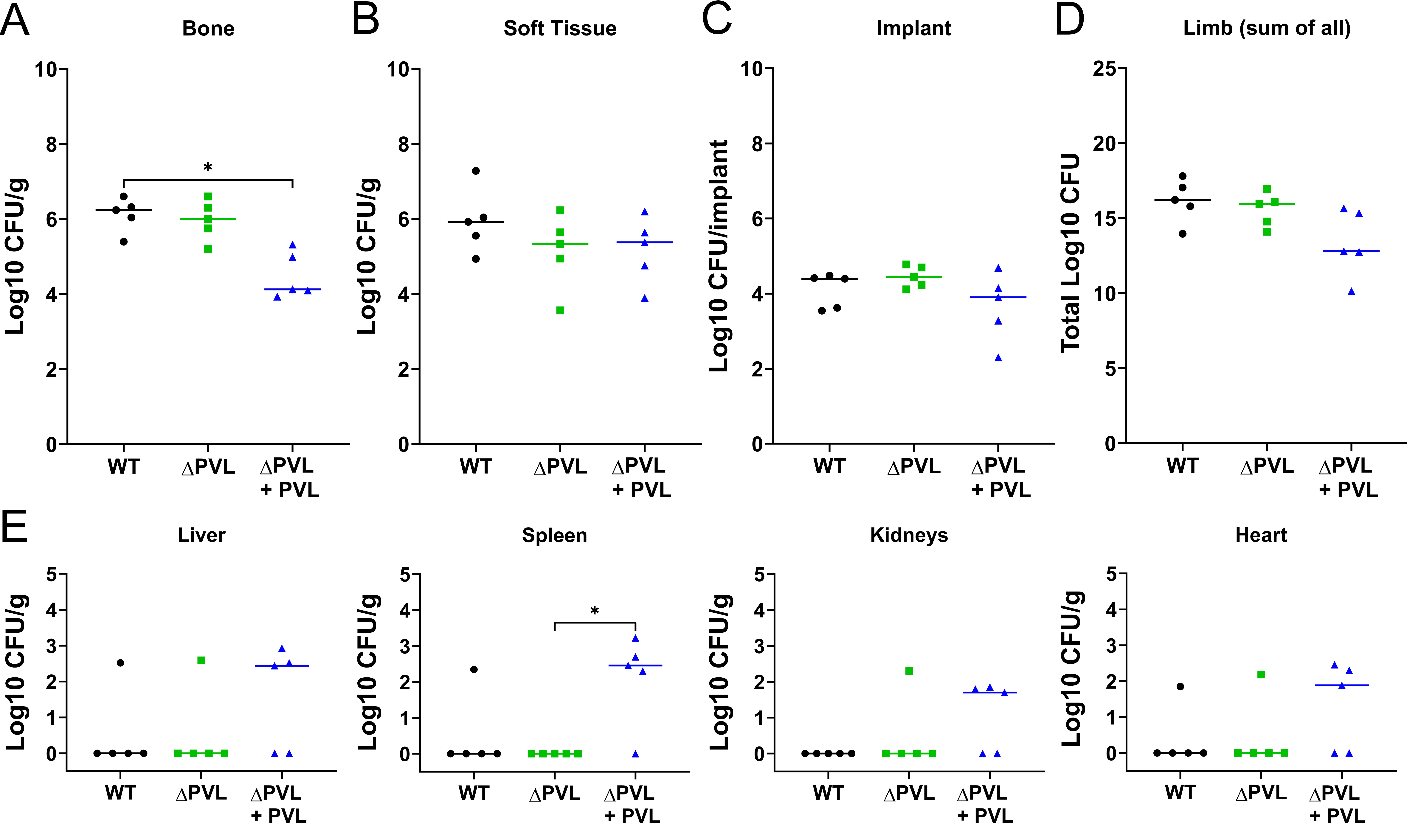

Supplement: Sup_Figure_3_Revised_2_ziad005 [file sup_figure_3_revised_2_ziad005.jpeg]

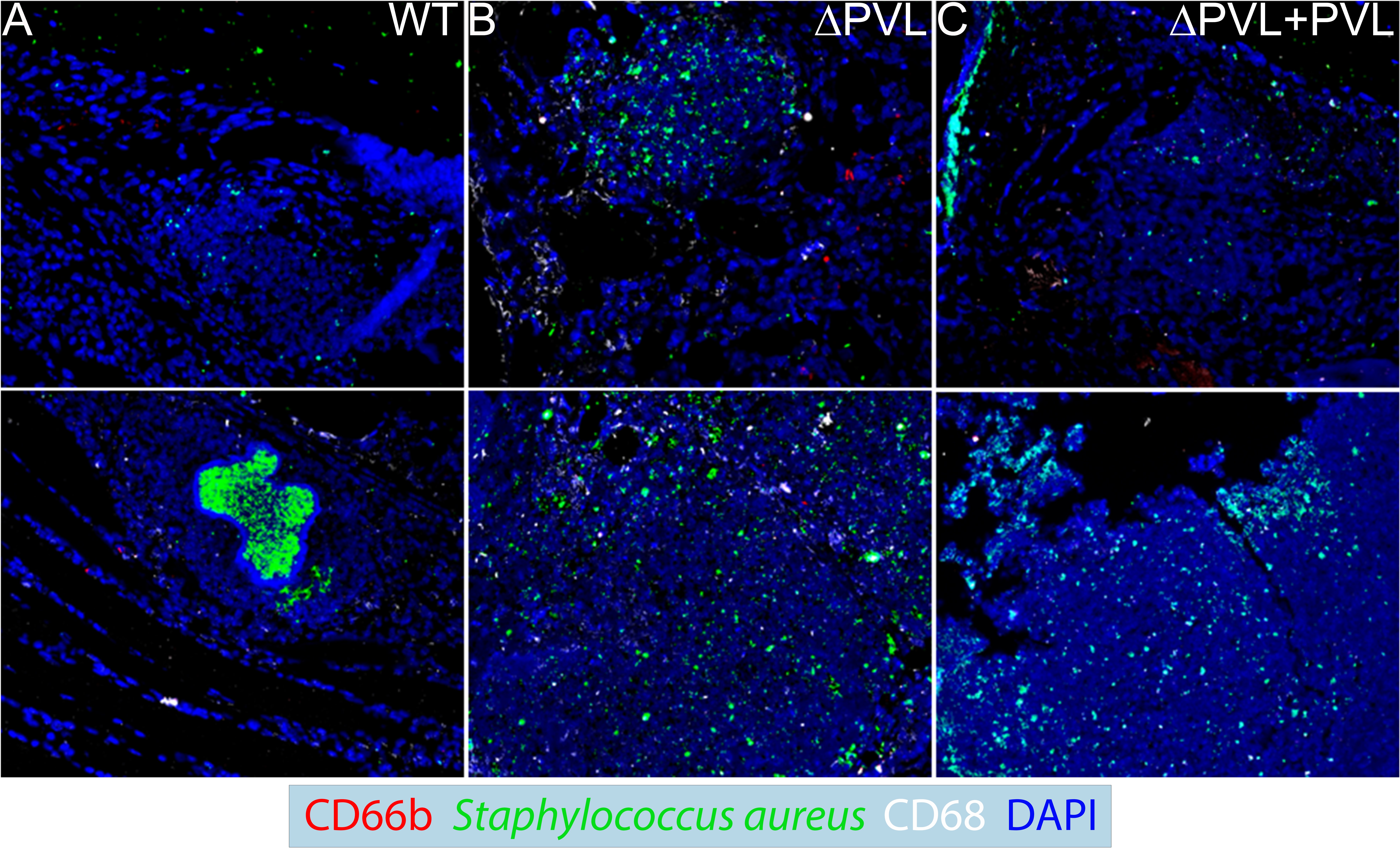

Supplement: Sup_Figure_4_2_ziad005 [file sup_figure_4_2_ziad005.jpeg]
